# Wider Access to Genotypic Space Facilitates Loss of Cooperation in a Bacterial Mutator

**DOI:** 10.1371/journal.pone.0017254

**Published:** 2011-02-25

**Authors:** Freya Harrison, Angus Buckling

**Affiliations:** Department of Zoology, University of Oxford, Oxford, United Kingdom; University of Zürich, Switzerland

## Abstract

Understanding the ecological, evolutionary and genetic factors that affect the expression of cooperative behaviours is a topic of wide biological significance. On a practical level, this field of research is useful because many pathogenic microbes rely on the cooperative production of public goods (such as nutrient scavenging molecules, toxins and biofilm matrix components) in order to exploit their hosts. Understanding the evolutionary dynamics of cooperation is particularly relevant when considering long-term, chronic infections where there is significant potential for intra-host evolution. The impact of responses to non-social selection pressures on social evolution is arguably an under-examined area. In this paper, we consider how the evolution of a non-social trait – hypermutability – affects the cooperative production of iron-scavenging siderophores by the opportunistic human pathogen *Pseudomonas aeruginosa*. We confirm an earlier prediction that hypermutability accelerates the breakdown of cooperation due to increased sampling of genotypic space, allowing mutator lineages to generate non-cooperative genotypes with the ability to persist at high frequency and dominate populations. This may represent a novel cost of hypermutability.

## Introduction

Bacterial populations readily evolve lineages with a 10–1000-fold increase in genomic mutation rate [1], [2]. In novel or changeable environments, adaptation is limited primarily by the supply of beneficial mutations and in these conditions ‘mutator’ genotypes can hitch-hike with advantageous mutations to reach high frequencies [3], [4], [5], [6], [7]. Mutator genotypes are common in bacterial infections, where pathogens are constantly challenged with a changing environment as a result of host immune responses and prophylactic intervention [8], [9], [10], [11]. However, when a population is well adapted to its environment, the increased rate of deleterious mutations in mutator lineages means that hypermutability is selectively disadvantageous [3], [4], [5], [12], [13]. It has also been suggested [14] that mutators may be selected against when population growth requires individuals to cooperate, as they are expected to generate cheating genotypes more readily and to decrease the relatedness [15] of evolving populations.

We have previously reported [16] that hypermutability accelerates the breakdown of cooperative behaviour in a bacterial species. Our model system for studying cooperation is the production of iron-scavenging siderophores by the opportunistic animal pathogen *P. aeruginosa*. Siderophores [17], [18], [19] are soluble molecules that are secreted by many microbial species in response to iron limitation; they facilitate growth in iron-limited environments by chelating iron, re-entering the bacterium via specific receptors and releasing their cargo of iron to cellular metabolism. Because iron is a major limiting factor for growth in aerobic environments and especially within animal hosts [20], [21], siderophores are important factors in determining bacterial growth and virulence [22], [23], [24] (see also [25]). As siderophores can be taken up by any cell with the cognate receptor, regardless of its own levels of siderophore production, they represent a public good: while individually metabolically costly to produce, siderophores confer a group-level benefit [22], [26]. Siderophore production may therefore be considered a form of cooperation between individual cells. As such, it is open to invasion by non-producing social “cheats,” which benefit from their neighbour's investment in siderophores while incurring no production costs of their own [16], [26], [27], [28], [29]. That these mutants are true cheats is demonstrated by the fact that their growth under iron limitation is enhanced by co-culturing with ancestral cooperators [22], [23]; that they can invade cooperating populations under conditions of local competition and low relatedness (but not high relatedness and global competition) [22] and that continuous variation in production of the primary siderophore, pyoverdin, is negatively correlated with their growth advantage over the ancestral cooperator in mixed culture [29]. The applicability of the *Pseudomonas*-siderophore system to the study of cooperation is evident from the number and range of social evolution hypotheses it has been used to test (for reviews see [30], [31]; for more recent work see [32], [33], [34]).

Using this model system, we have shown [16] that hypermutability facilitates the breakdown of siderophore cooperation when ecological conditions do not favour cooperation. Consistent with this, we have also shown [35] that mutator genotypes are selected against when ecological conditions confer inclusive fitness benefits on cooperation (high relatedness and inter-group competition: [15], [22]), and that this was attributable to their increased propensity to generate cheats. These observations are consistent with the established ability of mutator alleles to accelerate adaptation in novel environmental conditions [7], [36]. In both publications, we hypothesised that mutator lineages are able to show a more rapid adaptive response to the individual-level fitness benefits of cheating via two mechanisms. First, an increased mutation rate should lead to a simple increase in the rate at which cheating mutations are generated. Second, we suggested that our results may be partly attributable to the increased ability of mutator lineages to sample genotypic space and so generate genotypes that are only very rarely accessed by wild type lineages (see [6], [11] and references therein). Specifically, we suggested that mutator lineages might generate cheats better able to persist at high frequency in mixed cooperator/cheat populations. We now present a simple experiment that provides evidence in support of this hypothesis.

In our original study [16], wild-type and mutator (a *mutS* deletion mutant: [10]) populations of *P. aeruginosa* were passaged in pure culture for thirty days in iron-limited microcosms under entirely local competition, a condition known to select for the breakdown of cooperation [22]. Cheats increased in frequency more rapidly and reached higher maxima in mutator populations. Further, cheat frequencies showed a consistent increase over time in mutator populations, rising to dominance in all six replica populations; concomitantly, there was little variance in cheat dynamics between replica populations. This contrasted with wild-type populations, in which significant fluctuations in cheat frequency over time led to high between-replicate variation. We isolated individual clones from stored samples of these evolving populations and selected 18 mutator and 14 wild-type clones that produced <50% of the total siderophores produced by their cooperating ancestors (chromeazurol sulphate assay as in [16]). Competition experiments [37] were used to measure the relative fitness of each clone in competition with siderophore-producing bacteria at initially low (c. 5%) or high (c.50%) frequency.

## Results and Discussion

In order to test the hypothesis that the mutator genotype is more likely to produce cheats with a high fitness relative to the cooperative ancestral genotype, it is useful to ascertain whether the two strains differ in a) the variance in relative fitness among cheat clones and b) their median relative fitness.

**Figure 1:**
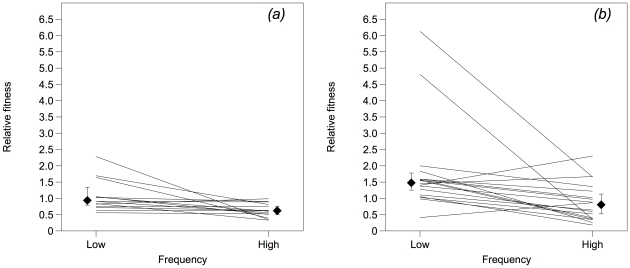
Relative fitnesses of cheat clones derived from the wild type (panel (a), n = 14) and mutator (panel (b), n = 18) populations described in [16]. Fitness was measured in competition with cooperating bacteria at initially low (c.5%) and high (c.50%) cheat frequency. Diamonds show medians and associated 95% confidence intervals.

As shown in Figure 1, both strains produce cheat clones that have a relative fitness >1 in the low-frequency condition. The larger variance in fitness among mutator-derived clones appeared to be driven by two outliers as the difference in variance was not statistically significant (Levene's test: test statistic = 1.21, *p* = 0.279). The median fitness of wild-type derived cheats was 0.94 and this was not significantly different from 1 (Wilcoxon signed rank test, H = 45, *p* = 0.660); the median fitness of mutator-derived cheats was 1.48 and this was significantly greater than 1 (H = 157, *p* = 0.001). Therefore, both genotypes produce cheats that can invade cooperating populations from rare and the range of relative fitnesses reachable by each ancestral genotype is similar.

Figure 1 clearly shows that there is a marked difference between strains with regard to high-frequency fitness; 14/14 wild-type-derived cheats have fitness <1 but 6/18 mutator-derived cheats have fitness >1. The variance among mutator-derived clones was significantly greater than among wild type-derived clones (Levene's test: test statistic = 8.33, *p* = 0.007). The median fitness of mutator-derived cheats was 0.81 and this was not significantly different from 1 (H = 60, *p* = 0.276) but the median fitness of the wild-type derived cheats, at 0.62, was significantly less than 1 (H = 0, *p* = 0.001). These results are consistent with the data previously reported [16] and support the hypothesis that mutator lineages are more likely to produce cheating genotypes which are able to persist at high frequencies in mixed culture with cooperators. Of course, we would predict that mutator lineages are also more likely to produce low-fitness genotypes (the ‘double-edged sword’ of mutation as outlined in our introduction), but these are presumably removed by purifying selection.

Our results are consistent with the general consensus that mutator lineages have increased responsiveness to selection pressure as a result of their ability to sample genotypic space more widely in a given time interval ([6], [11] and references therein). Our results are based on a relatively small sample of genotypes: this is due to our stringent condition for defining cheats as clones that produced less than 50% of the ancestral level of siderophore (for comparison, other authors have used a cut-off of 90% [29]). Nevertheless, our results provide an interesting observation of how this responsiveness can affect social behaviours in evolving populations: hypermutability leads to a rapid and more repeatable breakdown of cooperation under ecological conditions where it is not favoured This is consistent with observations that, conversely, hypermutability is selected against under ecological conditions that do favour cooperation [35].

Interestingly, there was no correlation between relative fitness at high and low frequency (GLM including strain and siderophore production relative to the ancestor; F_1,29_ = 0.68, *p* = 0.415), suggesting that mutations that confer better ability to invade from rare do not necessarily also confer a better ability to persist at high frequency. This observation suggests that, early in the period of evolution, the cheating mutants that we see are those that are able to gain a foothold in the population from an initially low frequency. In wild-type populations, once a certain proportion of cheats is reached it cannot be sustained as there is insufficient public good available and cheats decline, creating the ‘boom and bust’ cheat dynamics we described in the Introduction. In contrast, in mutator populations, the foothold established by these early cheats creates a niche for other cheat mutants which have little ability to invade cooperators from rare, but high ability to persist in a population where cheats are already common; in other words, ‘coloniser’ cheats are replaced by ‘persister’ cheats and a stable high frequency of cheats is maintained. The probability of mutations that generate ‘persister’ cheats occurring is presumably low enough that they are highly unlikely to arise in the absence of a significantly elevated mutation rate. A larger experiment would allow us to test this hypothesis explicitly by determining whether there is a correlation between the timepoint at which cheats were isolated from evolving populations and their relative fitness at high frequency. Furthermore, the genetic basis of the ‘persister’ ability attained by some mutator cheats remains to be dissected. It is possible that such genotypes may carry mutations that increase resilience to iron limitation.

Given the number of genes likely to affect siderophore production and uptake [17], [18], [19], [20], it is unsurprising that we see significant diversity in cheating phenotypes and fitness. This diversity may represent a range of different peaks on a rugged adaptive landscape, or different positions *en route* to a single peak. We cannot distinguish these alternatives with our data as it stands, but either way the mutator lineage seems to have acquired a range of cheating mutations with greater variability in fitness, and more high-fitness cheats, than the wild type. In the context of social evolution theory, it is rarely made explicit that a cheating phenotype could result from several different genotypes, potentially with different fitnesses relative to a cooperating phenotype. By showing that not all cheats are equal, our work underlines this point and raises interesting questions about the genetic bases of social traits and how these might affect the evolution of social behaviour. We therefore present our results as a set of intriguing observations that could inform new ways of looking at cooperator/cheat dynamics in evolving populations.

Additionally, both mutation rate and siderophore production have implications for bacterial virulence [8], [10], [16], [24], [38] and our study species is an important opportunistic human pathogen, causing significant morbidity and mortality among burns victims, intubated intensive-care patients and people with cystic fibrosis [39]. Oliver *et al* [9] report high frequencies of hypermutable *Pseudomonas aeruginosa* in cystic fibrosis patients. Further, it is known that siderophore non-producers accumulate in the lungs of cystic fibrosis patients as chronic infection progresses [28], [40], but it is not clear why this is the case (see [41] for a discussion of the evolutionary ecology of the CF lungs). Understanding the interplay between these traits may help to further our understanding of the intra-host ecology and evolution of chronic bacterial infections.

## Materials and Methods

Six glass tubes containing casamino acids (CAA) broth supplemented with 70 µg ml^−1^ human apotransferrin (Sigma Aldrich) and 20 mM sodium bicarbonate were inoculated with c. 10^6^ colony-forming units of wild type bacteria (PAO6049) and six with the same number of mutator bacteria (PAOΔ*mutS* [10]). PAO6049 is a methionine auxotroph and so the two strains may be distinguished in mixed populations by replica plating on CAA and M9 minimal agar. Populations were allowed to evolve for 30 days (c. 200 generations), with daily transfer to fresh medium. In mutator populations, cheats arose earlier, increased in frequency more rapidly and reached higher maximal frequencies than they did in wild-type populations. Mutator populations also showed less between-population variability in cheat frequency than did the wild-type populations (reproduced with permission from [16], see this publication for details of statistical tests). Cheats were isolated from these populations by plating aliquots of populations stored at −80°C on CAA agar and picking white colonies; the primary siderophore of this species, pyoverdin, fluoresces green in visible light and so white colonies represent potential non-producing siderophore “cheats.” Pyoverdin production has been shown to correlate well with total siderophore production [29].

The stored clones and their ancestors were assayed for siderophore production after 18 hours' growth in CAA+70 µg ml^−1^ human apotransferrin and 20 mM sodium bicarbonate at 37°C. The colorimetric chromeazurol sulphate assay was used as detailed in [16], [42] to determine siderophore production (total iron-binding capacity of supernatants) per colony-forming unit (CFU). We implemented a cut-off point for “cheating” such that only clones that produced siderophores at <50% of their ancestral level were used for further work. This left us with 14 wild-type-derived and 18 mutator-derived cheat clones. The distributions of % ancestral siderophore production were very similar: the first quartile, median, third quartile and range, respectively, were 11.25, 22.50, 40.00 and 45.00 for the wild-type cheats and 12.00, 27.50, 40.00 and 46.00 for the mutator cheats.

Each cheat clone was inoculated, along with the siderophore-producing ancestor of the oppositely-marked strain, into six replica microcosms containing 6 ml CAA+70 µg ml^−1^ human apotransferrin and 20 mM sodium bicarbonate. Densities were manipulated such that three microcosms contained c.5% cheats (low frequency condition) and three contained c.50% cheats (high frequency condition). Total starting density was c. 5×10^7^ CFU. The ancestral cooperator clones for each strain were competed against one another in exactly the same way. Relative fitness was calculated as described in [37] and each cheat's relative fitness was then expressed relative to its ancestor's fitness in the same condition. This gave a measure of each cheat's ability a) to invade a cooperating population from rare and b) to persist and proliferate at high frequency.

## References

[1] Sniegowski PD, Gerrish PJ, Johnson T, Shaver A (2000). The evolution of mutation rates: separating causes from consequences.. Bioessays.

[2] Sniegowski PD, Gerrish PJ, Lenski RE (1997). Evolution of high mutation rates in experimental populations of *E. coli*.. Nature.

[3] Bedau MA, Packard NH (2003). Evolution of evolvability via adaptation of mutation rates.. Biosystems.

[4] de Visser JA (2002). The fate of microbial mutators.. Microbiology.

[5] Giraud A, Matic I, Tenaillon O, Clara A, Radman M (2001). Costs and benefits of high mutation rates: adaptive evolution of bacteria in the mouse gut.. Science.

[6] Rainey PB (1999). Evolutionary genetics: The economics of mutation.. Curr Biol.

[7] Taddei F, Matic I, Godelle B, Radman M (1997). To be a mutator, or how pathogenic and commensal bacteria can evolve rapidly.. Trends Microbiol.

[8] Giraud A, Matic I, Radman M, Fons M, Taddei F (2002). Mutator bacteria as a risk factor in treatment of infectious diseases.. Antimicrob Agents Chemother.

[9] Oliver A, Canton R, Campo P, Baquero F, Blazquez J (2000). High frequency of hypermutable *Pseudomonas aeruginosa* in cystic fibrosis lung infection.. Science.

[10] Oliver A, Levin BR, Juan C, Baquero F, Blazquez J (2004). Hypermutation and the preexistence of antibiotic-resistant *Pseudomonas aeruginosa* mutants: implications for susceptibility testing and treatment of Chronic Infections.. Antimicrob Agents Chemother.

[11] Schaaff F, Reipert A, Bierbaum G (2002). An elevated mutation frequency favors development of vancomycin resistance in *Staphylococcus aureus*.. Antimicrob Agents Chemother.

[12] Funchain P, Yeung A, Stewart JL, Lin R, Slupska MM (2000). The consequences of growth of a mutator strain of *Escherichia coli* as measured by loss of function among multiple gene targets and loss of fitness.. Genetics.

[13] Trobner W, Piechocki R (1984). Selection against hypermutability in *Escherichia coli* during long term evolution.. Mol Gen Genet.

[14] Frank SA (1994). Kin selection and virulence in the evolution of protocells and parasites.. Proc Biol Sci.

[15] Hamilton WD (1964). The genetical evolution of social behaviour I & II.. J Theor Biol.

[16] Harrison F, Buckling A (2005). Hypermutability impedes cooperation in pathogenic bacteria.. Curr Biol.

[17] Cornelis P, Matthijs S (2002). Diversity of siderophore-mediated iron uptake systems in fluorescent pseudomonads: not only pyoverdines.. Environ Microbiol.

[18] Visca P, Imperi F, Lamont IL (2007). Pyoverdine siderophores: from biogenesis to biosignificance.. Trends Microbiol.

[19] Wandersman C, Delepelaire P (2004). Bacterial iron sources: from sideophores to hemophores.. Ann Rev Microbiol.

[20] Ratledge C, Dover LG (2000). Iron metabolism in pathogenic bacteria.. Ann Rev Microbiol.

[21] Payne SM (1993). Iron acquisition in microbial pathogenesis.. Trends Microbiol.

[22] Griffin AS, West SA, Buckling A (2004). Cooperation and competition in pathogenic bacteria.. Nature.

[23] Harrison F, Browning LE, Vos M, Buckling A (2006). Cooperation and virulence in acute *Pseudomonas aeruginosa* infections.. BMC Biol.

[24] Meyer JM, Neely A, Stintzi A, Georges C, Holder IA (1996). Pyoverdin is essential for virulence of *Pseudomonas aeruginosa*.. Infect Immun.

[25] Nyilasi I, Papp T, Takó M, Nagy E, Vágvölgyi C (2005). Iron gathering of opportunistic pathogenic fungi. A mini review.. Acta Microbiol Immunol Hung.

[26] West SA, Buckling A (2003). Cooperation, virulence and siderophore production in bacterial parasites.. Proc Roy Soc Lond B.

[27] Brown SP, Hochberg ME, Grenfell BT (2002). Does multiple infection select for raised virulence?. Trends Microbiol.

[28] de Vos D, de Chial M, Cochez S, Jansen B, Tümmler B (2001). Study of pyoverdine type and production by *Pseudomonas aeruginosa* isolated from cystic fibrosis patients: prevalence of type ii pyoverdine isolates and accumulation of pyoverdine-negative mutations.. Arch Microbiol.

[29] Jiricny N, Diggle SP, West SA, Evans BA, Ballantyne G (2010). Fitness correlates with the extent of cheating in a social bacterium.. J Evol Biol.

[30] Buckling A, Harrison F, Vos M, Brockhurst MA, Gardner A (2007). Siderophore-mediated cooperation and virulence in *Pseudomonas aeruginosa*.. FEMS Microbiol Ecol.

[31] Harrison F, Buckling A (2009). Cooperative production of siderophores by *Pseudomonas aeruginosa*.. Frontiers Biosci.

[32] Kümmerli R, Gardner A, West SA, Griffin AS (2009). Limited dispersal, budding dispersal and cooperation: an experimental study.. Evolution.

[33] Kümmerli R, Griffin A, West SA, Buckling A, Harrison F (2009). Viscous medium promotes cooperation in the pathogenic bacterium *Pseudomonas aeruginosa*.. Proc Roy Soc Lond B.

[34] Harrison F, Buckling A (2009). Siderophore production and biofilm formation as linked social traits.. ISME J.

[35] Harrison F, Buckling A (2007). High relatedness selects against hypermutability in bacterial metapopulations.. Proc Roy Soc Lond B.

[36] Tenaillon O, Toupance B, Le Nagard H, Taddei F, Godelle B (1999). Mutators, population size, adaptive landscape and the adaptation of asexual populations of bacteria.. Genetics.

[37] Lenski RE, Rose MR, Simpson SC, Tadler SC (1991). Long-term experimental evolution in *Escherichia coli*. I. Adaptation and divergence during 2,000 generations.. Am Nat.

[38] Racey D, Inglis RF, Harrison F, Oliver A, Buckling A (2010). The effect of elevated mutation rates on cooperation and virulence of *Pseudomonas aeruginosa*.. Evolution.

[39] Hauser AR, Rello J (2003). Severe infections caused by *Pseudomonas aeruginosa*.

[40] Smith EE, Buckley DG, Wu Z, Saenphimmachak C, Hoffman LR (2006). Genetic adaptation by *Pseudomonas aeruginosa* to the airways of cystic fibrosis patients.. Proc Natl Acad Sci U S A.

[41] Harrison F (2007). Microbial ecology of the cystic fibrosis lung.. Microbiology.

[42] Schwyn B, Neilands JB (1987). Universal chemical assay for the detection and determination of siderophores.. Anal Biochem.

